# Eight weeks of treatment with probiotic *Bifidobacterium breve*, Bif195 lowers fatigue scores in patients with diarrhoea-predominant irritable bowel syndrome: results from a randomised, clinical trial

**DOI:** 10.3389/fnut.2025.1701341

**Published:** 2026-01-21

**Authors:** Ida Marie Bruun Grønbæk, Sofie Ingdam Halkjær, Esben Holm Hansen, Sarah Mollerup, Sarah Juel Paulsen, Christina Valbirk Konrad, Sara Engel, Magdalena Bulinska-Balas, Anja Wellejus, Anne Birgitte Haaber, Alice Højer Christensen, Anne Line Engsbro, Andreas Munk Petersen

**Affiliations:** 1 Gastrounit, Medical Section, Copenhagen University Hospital – Amager and Hvidovre, Hvidovre, Denmark; 2 Department of Clinical Microbiology, Copenhagen University Hospital – Amager and Hvidovre, Hvidovre, Denmark; 3 Department of Clinical Medicine, University of Copenhagen, Copenhagen, Denmark; 4 Chr. Hansen A/S, Part of Novonesis, Human Health Biosolutions, Hørsholm, Denmark; 5 Mavespecialisten, Charlottenlund, Denmark; 6 Aleris Hospitals Copenhagen, Søborg, Denmark; 7 Department of Clinical Microbiology, Zealand University Hospital, Slagelse, Denmark

**Keywords:** *Bifidobacterium breve*, fatigue, gut microbiota, irritable bowel syndrome, probiotic treatment, shotgun metagenomics, transepithelial electrical resistance

## Abstract

Patients with irritable bowel syndrome experience abdominal pain and stool habit disturbances, and often also extraintestinal symptoms, such as fatigue. The disorder is linked to gut dysbiosis, and manipulation of the microbiota is considered a possible treatment strategy. This randomised, double-blinded, placebo-controlled study aimed to investigate the effects of the probiotic strain *Bifidobacterium breve*, Bif195™ (DSM 33360) (Bif195), on symptoms and gut microbiome composition in patients with diarrhoea-predominant irritable bowel syndrome. Sixty-one patients with moderate–severe disease activity were allocated to 8 weeks of treatment with either Bif195 or placebo (1:1), followed by 8 weeks of follow-up. The primary outcome was a change in symptom scores measured by the validated questionnaire, IBS-symptom severity scale. Secondary and explorative outcomes were the effects of Bif195 on intestinal symptoms, quality of life, fatigue, and the gut microbiota. Modulation of the transepithelial electrical resistance (TEER) of Caco-2 cells by Bif195 was investigated *in vitro* as a model of barrier integrity. The results showed no effect of Bif195 on primary or secondary outcomes; however, Bif195 lowered fatigue scores compared to placebo. Significantly increased TEER readings *in vitro* indicated enhanced barrier integrity, suggesting GI permeability as a mechanism for further clinical exploration.

**Clinical trial registration:**

clinicaltrials.gov, identifier NCT04808271.

## Introduction

Irritable bowel syndrome (IBS) is one of the most common gastrointestinal (GI) disorders worldwide, estimated to affect 3.8% of the population (with the Rome IV criteria), with large geographical variations (1).

The diagnosis is made when the IBS symptomatic criteria are met. Still, the patient is often also examined with no pathological findings on colonoscopy, faecal calprotectin (f-calprotectin) level, or stool testing for GI pathogens. In addition to the symptoms described in the diagnostic Rome IV criteria, about 50% of all IBS patients also suffer from extraintestinal symptoms, such as fatigue and psychological distress, with a higher prevalence among women (2). Combined with the limited treatment options, quality of life is often significantly impaired (2, 3).

The aetiology of IBS is not fully understood but is likely multifactorial (4). The disorder has been linked to dysbiosis of the gut microbiota (4, 5), and the altered microbiota may play a role in the pathogenesis by affecting gut permeability, immune responses, and neuromuscular functions (6). Especially diarrhoea, rather than constipation, is a symptom related to increased permeability and immune activation in the intestine (7).

Manipulation of the gut microbiota has been a focus point in the attempt to treat IBS with strategies such as faecal microbiota transplantation (FMT) and food supplementation with beneficial probiotic strains (8, 9). The perception has been that an effective probiotic treatment should contain many different bacterial strains to obtain better resilience; however, a recent systematic review of randomised controlled trials on the use of probiotic products, such as single and multiple probiotic strains, concluded that the choice of specific probiotic strains is likely more important for the efficacy than the number of strains (10). Possibly, tailored probiotic treatment based on the individual microbiome composition is the most successful approach.

*Bifidobacterium breve* (*B. breve*) is a lactic acid- and acetic acid-producing bacterial species naturally occurring in the healthy human intestine of both infants and adults, and it is found to be decreased in IBS patients (11). Previously, the probiotic strain *Bifidobacterium breve*, Bif195™ (DSM 33360), GALENEX™, in the following referred to as Bif195, was shown to protect against aspirin-induced mucosal damage in the stomach and small intestine of healthy subjects (12, 13).

This study aimed to investigate the effects of Bif195 on disease activity in patients with IBS-D by measuring IBS-related symptoms and quality of life. Further, we explored changes in the gut microbiome during 8 weeks of treatment and 8 weeks of follow-up. To explore the interaction with the gut barrier, we co-cultured Bif195 with mature Caco-2 monolayers *in vitro*.

## Materials and methods

### Clinical study design

**Figure 1:**
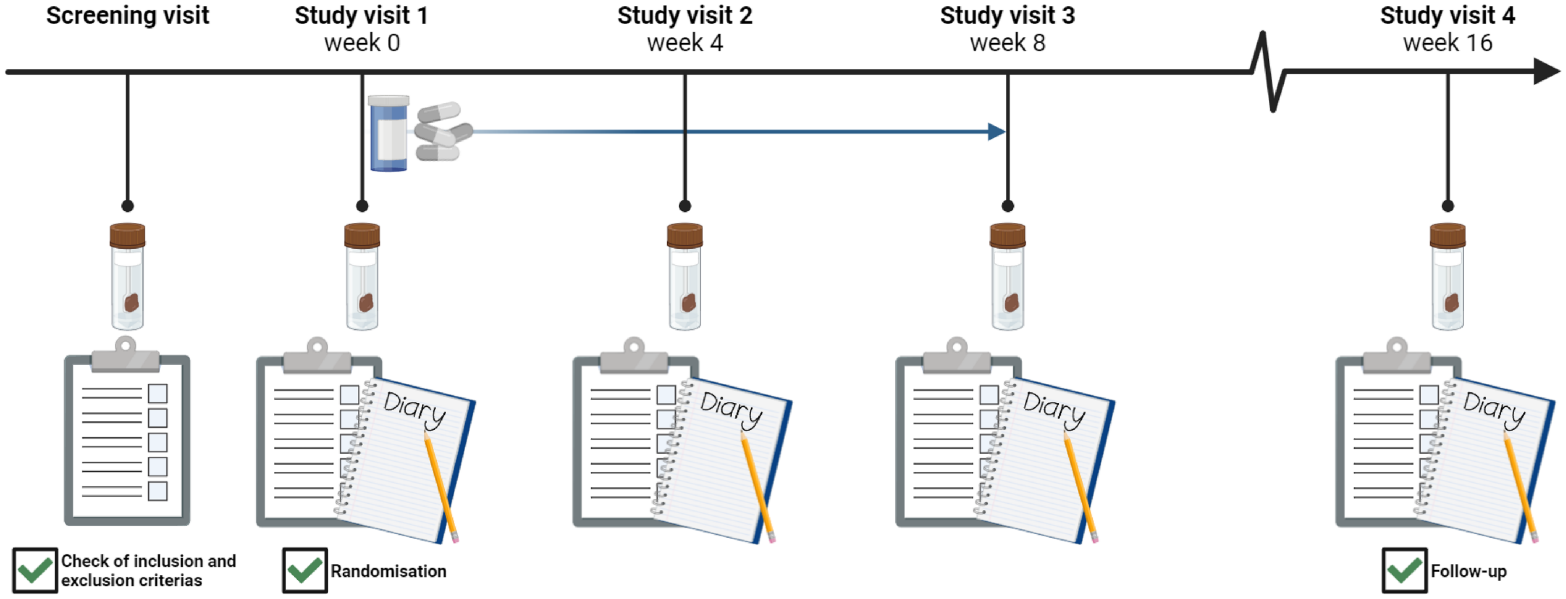
Study timeline with an illustration of the activities for all five visits.

The study was a randomised, double-blinded, placebo-controlled trial. Participants were invited for a screening visit, and if they met the inclusion criteria, they returned for study visit 1, where they were randomised. Participants were randomly assigned to treatment with either Bif195 or placebo (1:1) for 8 weeks, followed by an additional 8 weeks of follow-up. All included participants attended four study visits during the 16-week trial (Figure 1).

At all visits, participants delivered stool samples for gut microbiome analyses and completed two validated questionnaires regarding IBS-related symptoms and quality of life. For the 7 days preceding study visits 1–4, participants were instructed to keep a symptom diary of stool frequency, stool form, abdominal pain, and fatigue. Stool samples were collected in tubes without preservatives. Participants were asked to collect the sample on the same day as each study visit. If a toilet visit in the morning could not be guaranteed, stool samples could be collected the evening before and stored in the refrigerator (5 °C) until departure from home. All samples were frozen to −80 °C within 24 h and stored at this temperature until analyses were performed.

All study product was handed out at study visit 1, and participants were instructed to consume one capsule daily from study visit 1 to study visit 3. At study visits 2 and 3, after 4 and 8 weeks of intervention, respectively, the remaining capsules for each participant were counted by an investigator to assess compliance.

The aim was to include 60 participants based on the assumption that 40 and 80% of the participants in the placebo group and the Bif195 group, respectively, would achieve the primary endpoint criterion, with a power (1-*β*) of 0.8 and an alpha level of 0.05 (two-tailed test) for intention-to-treat analysis.

The trial was conducted in accordance with International Conference on Harmonisation E6 Good Clinical Practice, approved by the National Committee on Health Research Ethics, Capital Region of Denmark, and registered at clinicaltrials.gov (NCT04808271).

### Study participants

Participants were recruited between March 2021 and December 2022 via the outpatient clinic at the Gastrounit, Copenhagen University Hospital Hvidovre, Denmark. Participants were also recruited directly to the trial via referrals from private practitioners and/or hospitals and self-referrals. Information about the study was available locally at the recruitment sites and via advertisements on social media and in a patient-oriented magazine.

Participants were adults aged 18–70 years with IBS-D, fulfilling the ROME IV criteria for IBS, with moderate to severe disease activity (IBS symptom severity scale questionnaire [IBS-SSS] ≥ 175) (14). Furthermore, the following inclusion criteria had to be met: (1) ability to read and speak Danish, (2) given written informed consent, and (3) a normal colonoscopy within the last year on occurrence of bloody stool. The exclusion criteria were (1) history of other chronic gastrointestinal diseases and malabsorption diseases, e.g., ulcerative colitis, lactose intolerance, and coeliac disease, (2) f-calprotectin level >50 mg/kg, (3) abnormal biochemistry at screening visit, (4) consumption of antibiotics or probiotics within 4 weeks prior to inclusion, (5) positive finding of enteropathogenic microorganism, such as *Clostridioides difficile*, (6) surgical interventions in the GI region except for appendectomy, hernia repair, and gynaecological and urological procedures, (7) severe or not well-treated psychiatric disorders, (8) alcohol or drug abuse, (9) use of concomitant medication except contraceptives, hormone supplements, asthma/allergy agents, blood pressure agents, cholesterol-lowering agents, proton-pump inhibitors, non-prescription medicines, and psychiatric medicine (if the patient was well-treated, and the treatment had been stable for minimum 3 months and assessed by a physician not to be the cause of the IBS symptoms), and (10) pregnancy, planned pregnancy, or breast feeding.

Participants were instructed not to change their lifestyle throughout the study period.

### Study product description

Participants were instructed to take one capsule per day. The vegetable capsules used in the Bif195 and placebo groups were identical in appearance in terms of shape, size, colour, as well as, taste, and were packed in identical boxes. The random allocation sequence was generated by an independent party that was not otherwise involved in the trial. The allocation sequence consisted of blocks of various lengths, blinded to the parties involved in the study. A total of 80 boxes were labelled with individual randomisation numbers from 01 to 80, and the study product was handed out by a research investigator in chronological order.

The placebo capsules contained excipients only, whereas capsules for the Bif195 group also contained the probiotic Bif195 strain, amounting to a daily dose of at least 15 × 10^9^ colony-forming units (CFU). Product stability was monitored in parallel with the study.

Capsules were stored in a temperature-monitored refrigerator at the research site before use. Upon delivery, the study participants were instructed to store the capsules in their refrigerator at home.

### Outcomes and measures

The primary outcome of the study was a reduction in IBS symptoms after 8 weeks of treatment with Bif195 compared to placebo. Changes in IBS symptoms were assessed via the IBS-SSS questionnaire (14), which consists of five questions addressing the frequency and severity of abdominal pain, bloating, and impact on daily life for the past 10 days. Each question is scored from 0 to 100, with a total score ranging from 0 to 500 (0 = best, 500 = worst).

Secondary outcomes were differences in change between the Bif195 and placebo groups for quality of life, number of days with abdominal pain, abdominal pain score, and stool consistency. Changes in quality of life were based on the IBS Quality of Life questionnaire (IBS-QoL) (15, 16) containing 34 individually ranked items with a total score ranging from 0 to 100 (0 = best, 100 = worst). Abdominal pain was evaluated by each participant on an 11-point numeric rating scale (NRS) from 0 to 10 (0 = no pain, 10 = worst pain imaginable). The stool consistency was assessed using the Bristol Stool Chart (BSC) (17). The BSC is a validated scale designed to categorise the stool into seven different types of consistencies (type 1 = hardest, type 7 = softest). Types 3 and 4 are considered the optimal stool consistencies in general. Mean stool consistency was measured for each patient using the weighted stool score described in detail below.

Finally, the secondary outcome was the difference in the proportion of study participants reaching the IBS-SSS sum cut-off value after 8 weeks of treatment between the two groups. The cut-off value was defined as a reduction in the IBS-SSS sum of at least 50 points, as it is a frequently used threshold for a clinical response (18).

Explorative outcomes were differences between the Bif195 group and the placebo group regarding changes in fatigue scores, stool frequency, and urgency. Fatigue was evaluated by a single-item 11-point NRS from 0 to 10 (0 = no fatigue, 10 = worst fatigue possible) (19). Mean stool frequency was measured for each participant from information obtained in the BSC, see under weighted stool score. Urgency was registered by participants as a binary question, “Did you experience urgency today?” yes/no?

The effect of daily intake of Bif195 on the microbiome composition was also defined as an explorative outcome.

IBS-SSS, IBS-QoL, and the patient diary obtained at study visit 1 (i.e., at randomisation) were used as baseline data.

### Weighted stool score

Information about stool frequency and consistency obtained from the BSC in the patient diaries was converted into a numeric value using a weighted stool score generated by *Rubin et al.* (20, 21). Here, the stool types in the BSC were ranked from 0 to 3 depending on how much the consistency deviates from the normal (i.e., type 3 and 4): type 3 and 4 = 0 points, type 5 = 1 point, type 2 and 6 = 2 points, and type 1 and 7 = 3 points. The weighted stool score was calculated as an average of the daily stools. Example: 1 day with three stools of the BSC types 2, 4, and 7 results in a weighted stool score found by the formula x = (2 + 0 + 3)/3.

One day without stool is awarded 1 point, while consecutive days without stool are awarded points corresponding to the exact number of days without bowel movement.

If a study participant had categorised a stool as multiple BSC types, then the average of the highest and the lowest scores was calculated.

### Microbiome analyses and QPCR

DNA for gut microbiome analyses was extracted and sequenced, and the data were analysed as previously described by Grønbæk et al. (22). Differences in richness and Shannon diversity between week 0 and week 8 within the Bif195 and placebo groups were tested using the Wilcoxon signed-rank test. Differences in beta diversity between week 0 and week 8 within the two groups were tested with PERMANOVA using the adonis2 function of the R package vegan v. 2.6.6.1 (23) and the permute package v. 0.9.7 to define restricted permutations (setting blocks to patient ID as the samples are paired). Differences in beta diversity at week 0 depending on the fatigue level and the IBS-SSS score were also assessed. The fatigue scores were divided into four groups: 0–2, >2–4, >4–6, and >6, and IBS-SSS scores were evenly divided into four groups in chronological order. Differences among these groups were tested using adonis2 for the two individual scores separately. Methods for differential abundance analyses comparing week 0 and week 8 have also been previously described by Grønbæk et al. (22).

Bif195-specific quantitative PCR (QPCR) was performed on a LightCycler 480 (Roche) using Takyon™ No Rox Probe MasterMix dTTP (Eurogentec). The final PCR contained 1 ng/μL DNA, 300 nM forward and reverse primers, and 150 nM probe. Forward primer: CCTCGTCCGTTTACCTTTCA; reverse primer: CAAAACAAGCGTCCACAAAA; probe: TCATTGTTCTACTTGGTGCAAGA. PCR programme: 50 °C 2 min, 95 °C 10 min (95 °C 15 s, 60 °C 1 min) × 40.

### *In vitro* study

#### *Bifidobacterium breve* Bif195 cultivation

Bif195 was inoculated from a frozen ampoule from the Chr. Hansen Culture Collection (CHCC) in Man-Rogosa-Sharpe pH 6.5 (MRS, BD Difco, NY, United States) medium, supplemented with w/v 0.05% L-cysteine-HCl (Merck, KGaA, Darmstadt, Germany). The strain was incubated under anaerobic conditions with AnaeroGen sachets (Thermo Scientific, Oxoid, MA, United States) overnight at 37 °C. A 10-fold dilution series was prepared from the overnight culture and incubated under the same conditions as described above. Bacterial cells from late exponential/early stationary phase were harvested by centrifugation (3,500 *g* for 10 min), washed twice with pre-warmed Hanks’ Balanced Salt Solution (HBSS, Gibco, 14175, Darmstadt, Germany), and resuspended in Dulbecco’s Modified Eagle Medium (DMEM, Gibco, 21885, Darmstadt, Germany) medium for further investigation.

#### Caco-2 cell culture

Human colon adenocarcinoma (Caco-2) cells were obtained from the DSMZ ACC 169. Cells were routinely cultured in 75-cm^2^ flasks at 37 °C in a 5% CO_2_ humidified environment, with the medium replaced every 2–3 days. Caco-2 cells were grown in DMEM (Gibco, 21885, Darmstadt, Germany) containing 10% heat-inactivated foetal calf serum (FBS, Gibco 10500–064, Merck, Darmstadt, Germany), 1% nonessential amino acids, 100X (BioWest, X0557, Nuaillé, France), and Penicillin–Streptomycin–Amphotericin B solution (Biological Industries 03-033-1B, Connecticut, United States). Cells were sub-cultivated at 80% confluence by adding TrypLE™ (Gibco 12604 Merck, Darmstadt, Germany).

#### TEER assay

For the transepithelial electrical resistance (TEER) assay, Caco-2 cells were seeded on 12-well semi-permeable polyester membranes (Transwell™ Corning Cat #3460, Merck, Darmstadt, Germany) at a density of 10^5^ cells/ml and cultured for 21 days to obtain differentiated cell monolayers. The cell media were replenished twice per week in both apical and basolateral compartments.

The day prior to bacterial exposure, the cell media were replaced with antibiotic-free media, and the seeded Transwell™ inserts were transferred to the CellZscope2 device (NanoAnalytics GmbH, Münster, Germany). The CellZscope2 was placed in a water-saturated incubator at 37 °C and 5% CO_2_, and hourly TEER measurements were initiated to generate baseline TEER readings for each well. On the day of the experiment, the impact of Bif195 on barrier integrity was studied by replacing 100 μL of apical medium with the live bacterial suspension (final OD_600nm_ 0.5). DMEM media served as a negative control, and the experiment was performed in triplicate.

The TEER was measured as *Ω* per cm^2^, and the change in TEER was calculated as a percentage change relative to baseline recordings immediately before adding either bacterial solution or control media.

### Statistical analyses

The statistical methods for the microbiome analyses are described under the headline “Microbiome data analyses.” For the clinical study, statistical analyses were performed with the statistical software SAS® Release 9.3 or later versions by the independent, external firm Signifikans ApS, Denmark. The study was blinded until after the statistical analyses were performed.

For the secondary endpoint analysis of the difference in the proportion of participants reaching the IBS-SSS sum cut-off value, a chi-squared test was used. For all other primary, secondary, and explorative endpoints, the differences between the Bif195 group and the placebo group were assessed by ANCOVA analysis of the delta values (change at 8 weeks from baseline) with baseline values as covariate. Baseline characteristics with comparisons between groups were done by the chi-squared test for categorical variables and Student’s *t*-test for continuous variables.

For statistical analyses, participants were grouped as the full analysis set (FAS) and per-protocol analysis set (PPS), excluding participants with records of major protocol deviations. PPS will be reported unless the other is described. All participants met the criteria for FAS, and therefore, safety data will be reported on the FAS population.

For the *in vitro* TEER AUC results, differences between the groups were tested by an unpaired *t*-test using GraphPad Prism (version 10.3.1, GraphPad Software).

## Results

### Patient characteristics

**Figure 2:**
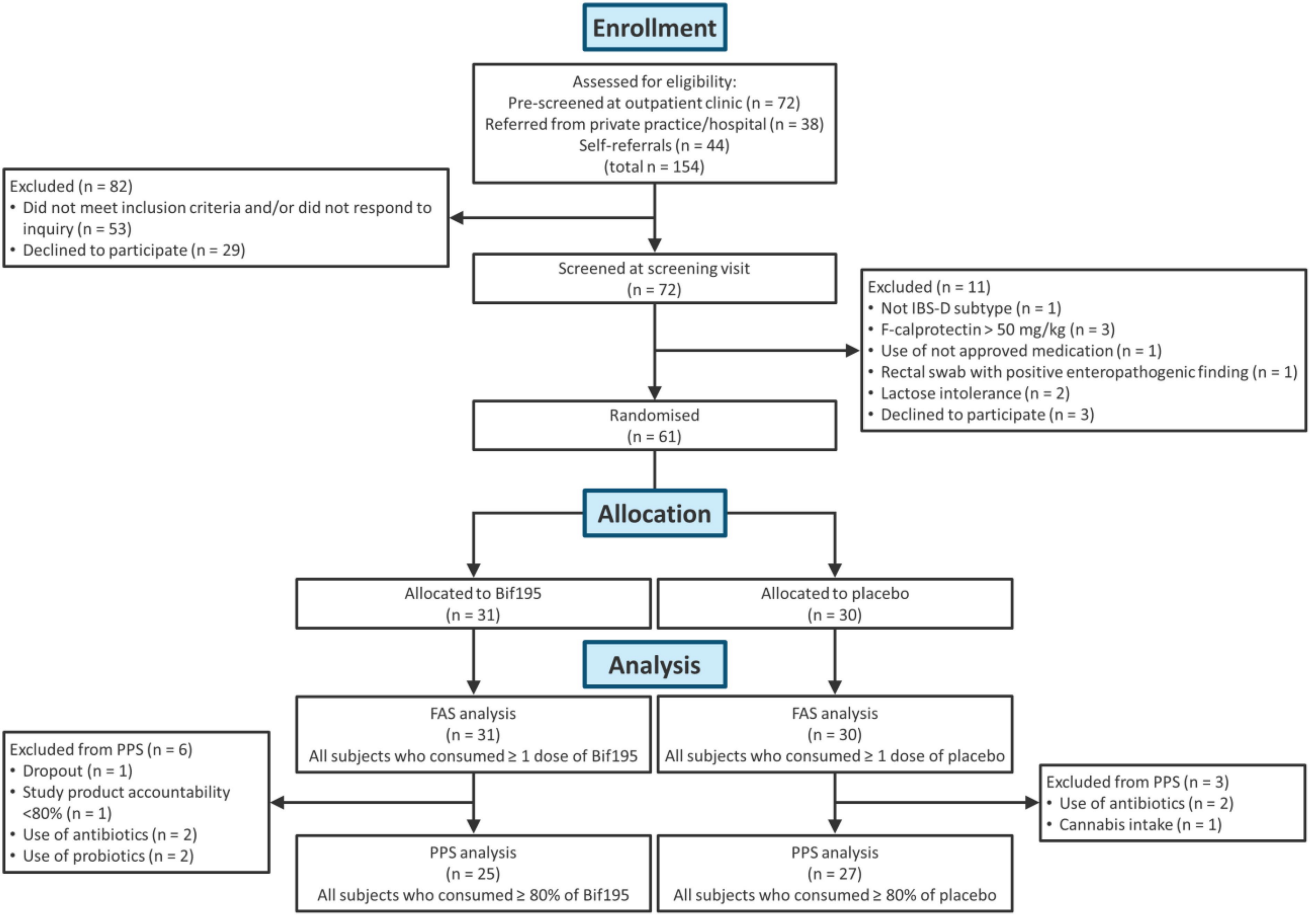
Flowchart of patients who were included and excluded from analysis. Full analysis data set (FAS), per-protocol data set (PPS).

Seventy-two patients consented to participate in the study, and of these, 61 participants met the inclusion criteria and were included in the trial (Figure 2).

31 participants were allocated to treatment with Bif195, and 30 to treatment with a placebo. A total of nine participants were excluded from the PPS due to dropout (*n* = 1) and major protocol deviations, such as the use of antibiotics (*n* = 4) or probiotics (*n* = 2) during the intervention period, cannabis intake (*n* = 1), and a study product consumption <80% (*n* = 1).

Baseline characteristics of participants are presented in Table 1. Participants were older in the placebo group compared to the Bif195 group [mean (SD); 37.2 (13.6) vs. 29.6 (9.1), respectively, *p* = 0.022]. In addition, approximately two-thirds of the participants were women in the placebo group, whereas only half were women in the Bif195 group.

**Table 1:** Baseline characteristics

Characteristics	Overall (*n*=52)	Bif195 (*n*=25)	Placebo (*n*=27)
**Age [mean (SD)]**	33.5 (12.2)	29.6 (9.1)	37.2 (13.6)
**Sex, *n* (%)**			
Male	22 (42)	13 (52)	9 (33)
Female	30 (58)	12 (48)	18 (67)
**BMI [mean (SD)]**	25.2 (4.6)	24.4 (4.3)	26.0 (4.9)
**Medication, *n* (%)**	46 (89)	23 (92)	23 (85)
Contraceptives	14 (27)	7 (28)	7 (26)
Hormone supplements	2 (4)	1 (4)	1 (4)
Asthma/allergy agents	10 (19)	6 (24)	4 (15)
Blood pressure agents	5 (10)	0	5 (19)
Cholesterol-lowering agents	2 (4)	0	2 (7)
Proton pump inhibitors	7 (14)	3 (12)	4 (15)
Antidepressants	1 (2)	1 (4)	0
Peristalsis regulating agents	8 (15)	4 (16)	4 (15)
Vitamin and nutritional supplements	15 (29)	6 (24)	9 (33)
Other	17 (33)	7 (28)	10 (37)
**Alcohol intake (units/week), *n* (%)**			
0	18 (35)	8 (32)	10 (37)
1-7	27 (52)	13 (52)	14 (52)
8-14	6 (12)	3 (12)	3 (11)
>14	1 (2)	1 (4)	0
**Smoking habits, *n* (%)**			
Active smoker	17 (33)	7 (28)	10 (37)
Not smoker	35 (67)	18 (72)	17 (63)
**IBS-SSS score at screening visit [mean (SD)]**	342.8 (72.4)	331.9 (67.9)	353.0 (76.3)
**IBS-SSS score at baseline (study visit 1) [mean (SD)]**	331.8 (71.7)	318.5 (63.9)	344.1 (77.5)
**IBS-QoL score at screening visit [mean (SD)]**	47.0 (18.4)	46.4 (18.0)	47.6 (19.2)
**IBS-QoL score at baseline (study visit 1) [mean (SD)]**	44.3 (18.7)	42.2 (18.7)	46.2 (18.9)

*n*, number of subjects within the population; %, percent of subjects in the study. IBS-SSS, IBS-symptom severity scale questionnaire; IBS-QoL, IBS-quality of life questionnaire; SD, standard deviation. IBS-SSS score range: 0–500 (0 = best, 500 = worst). IBS-QoL score range: 0–100 (0 = best, 100 = worst).

There were no statistically significant differences in the gut microbiome between the two groups at baseline (data not shown).

### Primary outcome: IBS symptoms

There were no statistically significant differences between the Bif195 group and the placebo group in improvements in IBS symptom scores after 8 weeks of treatment (Table 2). Furthermore, within the individual groups, there were no statistically significant changes from baseline and after 4 or 8 weeks of intervention, or after 8 weeks of follow-up (data not shown).

**Table 2:** Primary and secondary outcomes at baseline, after 8 weeks, and change from baseline

Outcomes	Overall (*n*=52)	Bif195 (*n*=25)	Placebo (*n*=27)	* *p-value*
Primary outcome				
**IBS-SSS score, mean (SD)**				
Baseline	331.8 (71.7)	318.5 (63.9)	344.1 (77.5)	0.611
After 8 weeks	291.2 (94.0)	275.2 (91.8)	305.9 (95.3)	
Change from baseline	−40.7 (77.2)	−43.3 (79.7)	−38.2 (76.1)	
Secondary outcomes				
**IBS-QoL score, mean (SD)**				
Baseline	44.3 (18.7)	42.2 (18.7)	46.2 (18.9)	0.374
After 8 weeks	39.5 (19.6)	36.3 (18.9)	42.5 (20.1)	
Change from baseline	−4.8 (11.5)	−5.9 (12.8)	−3.7 (10.3)	
**Number of days with abdominal pain, mean (SD)**				
Baseline	6.7 (2.9)	6.1 (2.8)	7.3 (2.9)	0.534
After 8 weeks	6.1 (3.2)	6.0 (3.1)	6.1 (3.3)	
Change from baseline	−0.6 (3.0)	−0.1 (2.5)	−1.1 (3.4)	
**Abdominal pain score, mean (SD)**				
Baseline	56.1 (20.9)	52.2 (22.8)	59.8 (18.6)	0.755
After 8 weeks	46.9 (21.9)	44.4 (21.0)	49.2 (22.9)	
Change from baseline	−9.3 (23.8)	−7.8 (23.6)	−10.6 (24.4)	
**Stool consistency (weighted stool score), mean (SD)**				
At baseline	1.3 (0.5)	1.3 (0.6)	1.4 (0.5)	0.387
After 8 weeks	1.1 (0.6)	1.0 (0.6)	1.2 (0.6)	
Change from baseline	−0.2 (0.5)	−0.2 (0.5)	−0.1 (0.5)	
**Participants with significant improvement in IBS symptoms**				
≥50 cut-off, *n* (%)	29 (55.8)	14 (56.0)	15 (55.6)	0.705

*n*, number of subjects within the population; %, percent of subjects in the study. Cut-off, 50 points reduction in IBS-SSS sum after 8 weeks of treatment. IBS-SSS, IBS-symptom severity scale questionnaire; IBS-QoL, IBS-quality of life questionnaire; SD, standard deviation.

A decrease in IBS-SSS score, IBS-QoL score, abdominal pain score, and weighted stool score is considered an improvement in symptoms. * ANCOVA analysis of the mean delta values (V3-V1), with baseline values as covariate, chi-squared test was used for categorical variables.

Due to differences in baseline characteristics between the groups, statistical adjustments for sex and age (participants divided into ≤50 years and >50 years) were performed, but it did not change the overall outcome.

### Secondary outcomes: quality of life, number of days with abdominal pain, abdominal pain score, stool consistency, and IBS-SSS sum cut-off value

None of the secondary outcomes were statistically significantly different between the Bif195 group and the placebo group (Table 2). Adjustments for sex and age on secondary outcomes did not change the outcome of the results.

### Explorative outcomes: fatigue, stool frequency, and urgency

**Figure 3:**
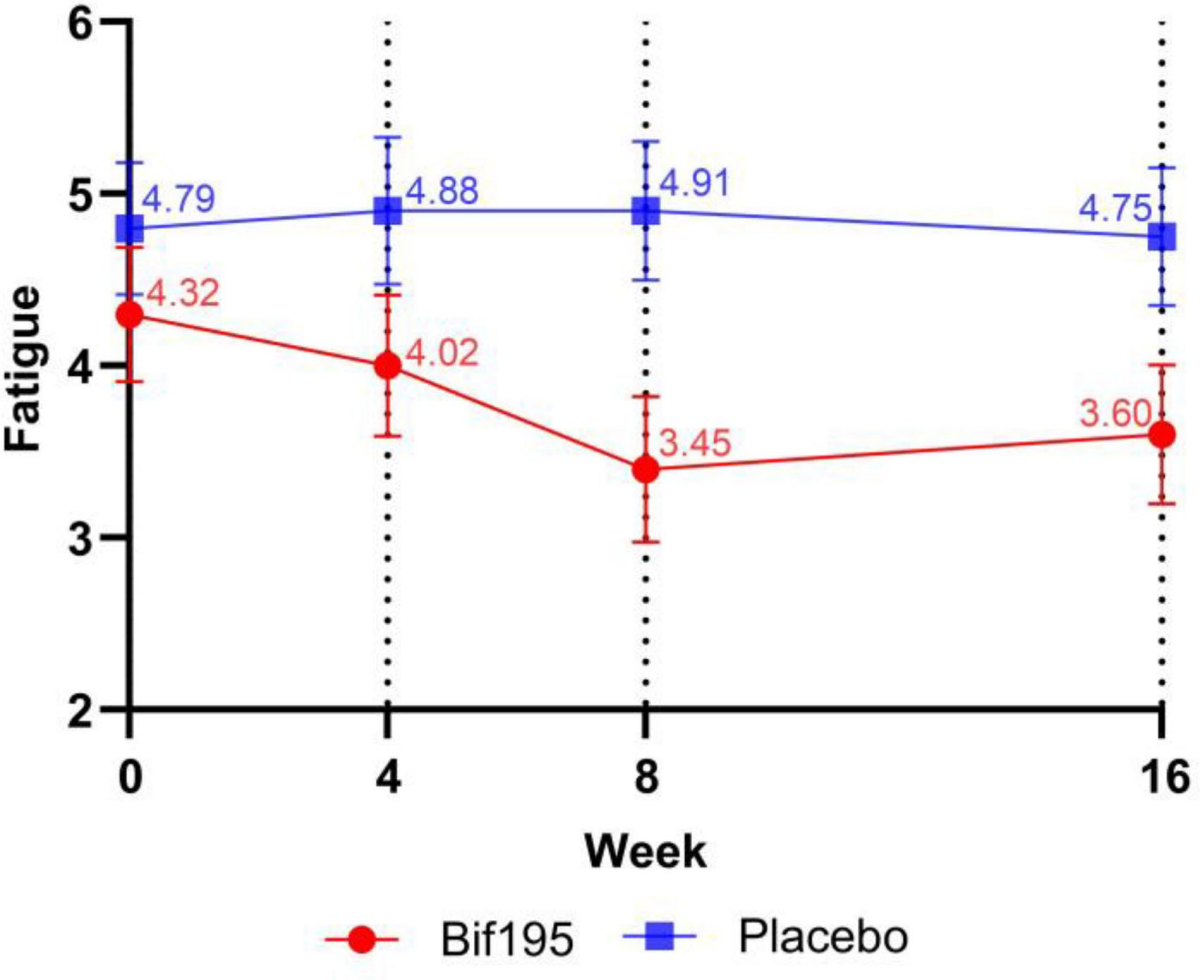
Fatigue scores in the Bif195 group and placebo group at week 0 (baseline), 4 weeks (halfway through intervention), 8 weeks (end of intervention), and 16 weeks (follow-up). All values are mean ± SD. *n* = 51 at 4 weeks and *n* = 52 at all other timepoints. Statistical analyses were an ANCOVA analysis of the mean delta values, with baseline values as covariates and adjustment for sex and age. There were statistically significant differences in change in fatigue scores between the groups after 8 weeks (*p* = 0.011), but not after an additional 8 weeks of follow-up (*p* = 0.066).

We found that participants in the Bif195 group had a significantly larger drop in fatigue scores after 8 weeks of treatment (thus feeling less fatigue) compared to the placebo group, where scores remained unchanged (Figure 3; Supplementary Table 1). When adjusted for sex and age, the difference in change remained statistically significant (*p* = 0.011). At follow-up 8 weeks after termination of treatment, the change in mean fatigue score was no longer significantly different between the groups [mean (SD) Bif195 vs. placebo; −0.7 (1.53) vs. 0.0 (1.51), *p* = 0.066, adjusted for sex and age].

There were no differences between the groups in change in stool frequency or urgency during the 8 weeks of intervention (Supplementary Table 1). However, when adjusting for sex and age, there was an improvement in urgency in the placebo group compared to the Bif195 group in the FAS [mean (SD); −0.5 (1.00) vs. 0.2 (1.54), respectively, *p* = 0.035, FAS], but not in PPS [mean (SD); −0.5 (1.05) vs. 0.3 (1.67), respectively, *p* = 0.066, PPS].

### The gut microbiome

**Figure 4:**
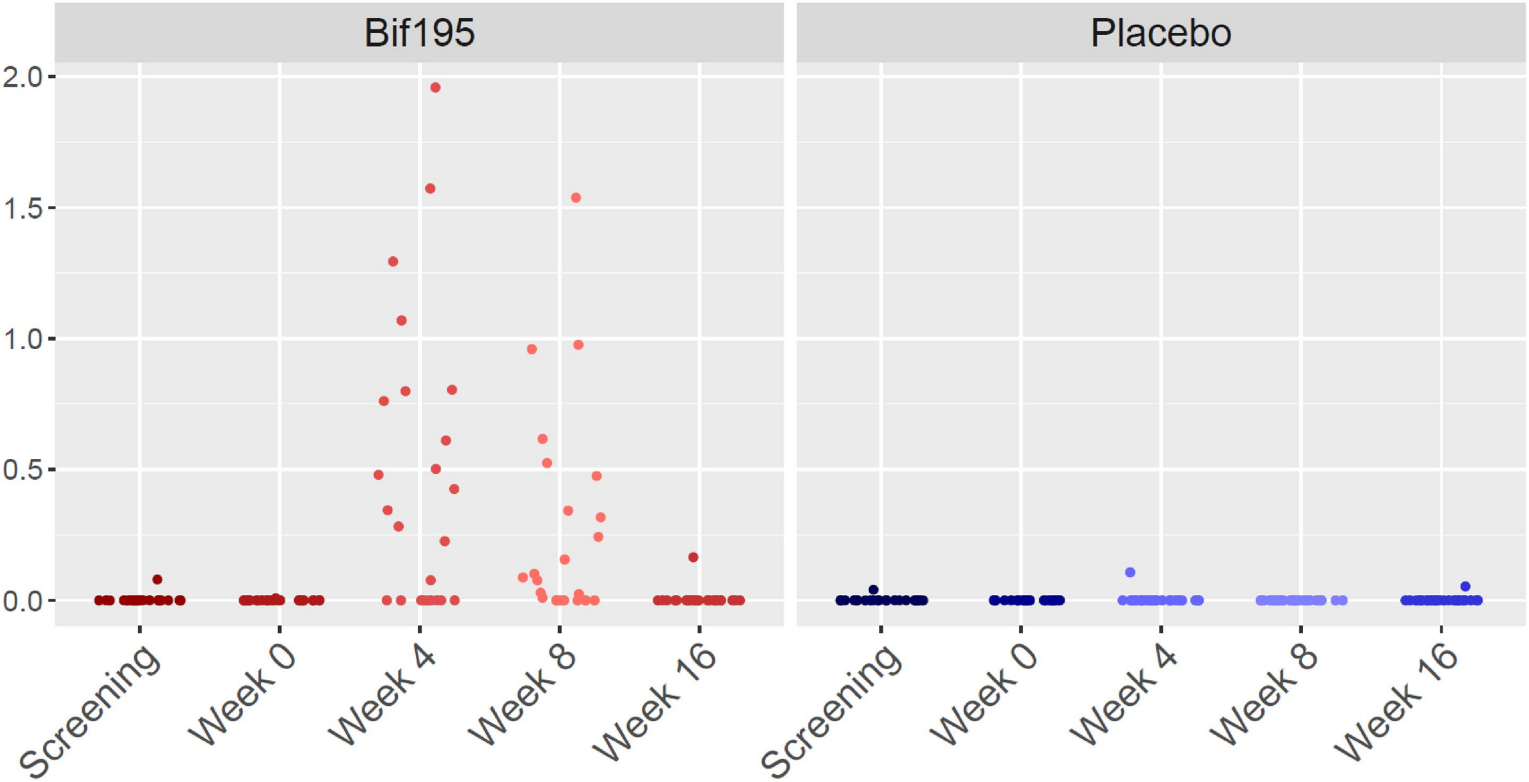
Relative abundance (%) of *Bifidobacterium breve* divided into study visits in the Bif195 group and the placebo group. In the Bif195 group, *Bifidobacterium breve* was significantly more abundant after 8 weeks of treatment compared to baseline at week 0, *p* = 0.027.

We investigated the microbiome composition and differentially abundant bacterial species at each study visit in the two groups. In the Bif195 group, *B. breve* was the only differentially abundant species after the 8 weeks of intervention compared to baseline at week 0. The difference was, however, only significant for one of the two tests when adjusting for multiple testing [Bif195 group week 0 vs. week 8, *p* = 0.027 (Benjamini–Hochberg corrected *p*-value of Welch’s *t*-test), *p* = 0.06 (Benjamini–Hochberg corrected p-value of Wilcoxon test), and effect size 0.973] (Figure 4). After 8 weeks without treatment (follow-up at week 16), the relative abundance of *B. breve* had returned to baseline levels, indicating that *B. breve* did not engraft robustly in the host’s microbiota during the 8 weeks of intervention. No species were differentially abundant between the baseline visit and after 8 weeks of intervention in the placebo group.

**Figure 5:**
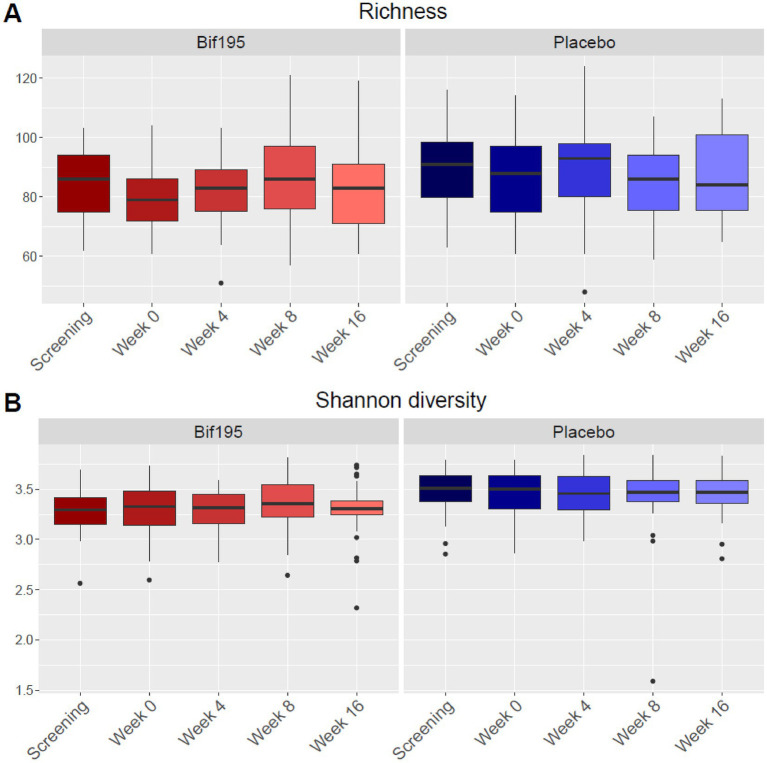
Alpha diversity with richness **(A)** and Shannon diversity **(B)** divided into study visits in the Bif195 group and the placebo group. In the Bif195 group, richness was significantly increased after 8 weeks of treatment compared to baseline at week 0, *p* = 0.031.

Alpha diversity is illustrated in Figures 5A,B. For the Bif195 group, richness was statistically significantly increased after 8 weeks of treatment compared to baseline (*p* = 0.031) and not in the placebo group (*p* = 0.81). There were no differences in Shannon diversity between baseline and after 8 weeks of treatment within any of the groups.

Beta diversity was significantly different between baseline and after 8 weeks of treatment in the Bif195 group (*p* = 0.015). This was not the case in the placebo group (*p* = 0.786) (Supplementary Figure 1).

After discovering that Bif195 affects fatigue scores, we performed a *post hoc* analysis investigating potential correlations between fatigue scores and the gut microbiome at baseline. We also investigated potential correlations between the baseline gut microbiome and IBS–SSS scores. No correlations between the microbiome and the fatigue scores or the IBS–SSS scores were found (data not shown).

By qPCR analyses, Bif195 DNA was found in stool samples from all participants allocated to Bif195 treatment. In 25/30 of the participants in the Bif195 group (drop-out in the Bif195 group not included in the analysis), Bif195 DNA was detected in stool samples at both week 4 and week 8, but not at follow-up at week 16. In 4/30 participants, Bif195 DNA was found only at week 4 or week 8. Only in one participant, Bif195 DNA was detected at week 4, 8, and 16.

### Safety and adverse events

Fifteen adverse events were registered and divided into eight categories (Table 3). No adverse events were more prominent in one group compared to the other; however, there was a tendency for more participants to report bloating and/or flatulence in the placebo group compared to the Bif195 group. Nine events were reported as mild in intensity, three were moderate, and three were severe in intensity. Events were assessed as related to treatment (yes/no). Of the six events assessed to be related to the treatment, two were reported from the Bif195 group and four from the placebo group. The two reported events from the Bif195 group were abdominal pain and skin rash. No serious adverse events were reported.

**Table 3:** Adverse events

Events	Overall		Bif195 (*n*=31)	Placebo (*n*=30)	* p-value
	E	(*n*=61)			
All adverse events	15	13 (21.3)	7 (22.6)	6 (20.0)	
Abdominal pain	2	2 (3.3)	2 (6.5)	0	0.157
Diarrhoea	1	1 (1.6)	1 (3.2)	0	0.321
Constipation	4	4 (6.6)	3 (9.7)	1 (3.3)	0.317
Bloating and/or flatulence	3	3 (4.9)	0	3 (10.0)	0.071
Nausea	2	2 (3.3)	0	2 (6.7)	0.144
Borborygmi	1	1 (1.6)	0	1 (3.3)	0.305
Skin rash	1	1 (1.6)	1 (3.2)	0	0.321
Other	1	1 (1.6)	0	1 (3.3)	0.305

*n*, number of subjects within the population. E, number of events. %, percent of subjects in the study. Other, bilateral breast pain. * Chi-squared test is used for categorical variables.

### Transepithelial electrical resistance (TEER) response induced by *Bifidobacterium breve*, Bif195

**Figure 6:**
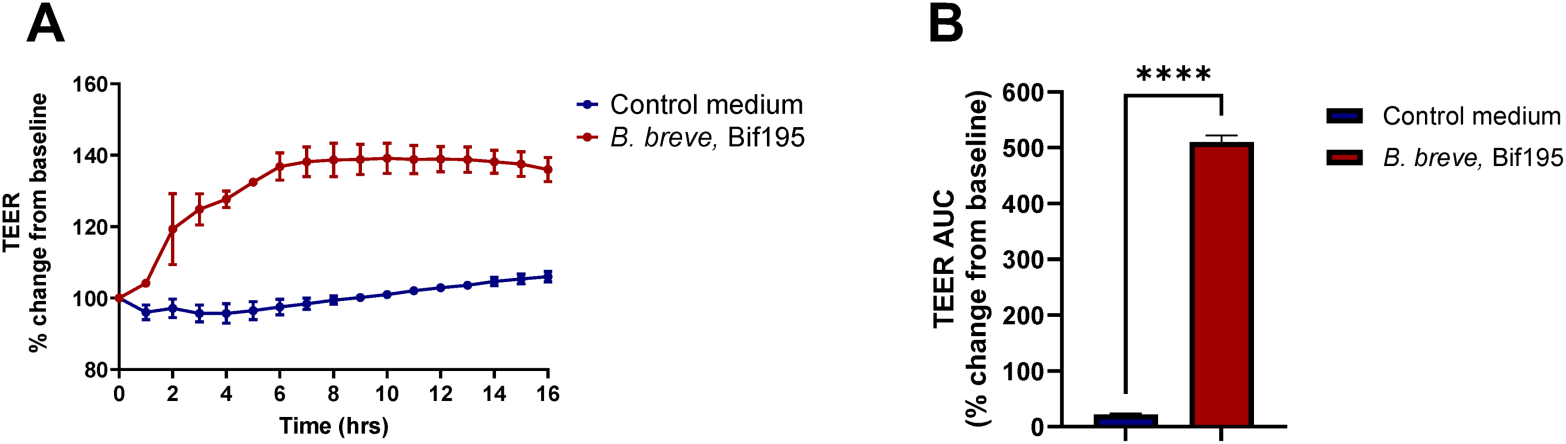
Modulation of barrier integrity *in vitro*. Transepithelial electrical resistance (TEER) measurements were performed on co-cultures of matured Caco-2 monolayers with Bif195 or medium control over 16 hours **(A)** and percentage change from baseline was calculated **(B)**. TEER AUC was calculated per well and differences between the two groups were tested via an unpaired *t*-test. Values are mean ± SD.

In an *in vitro* assay 21-day-old differentiated Caco-2 cells were co-incubated with Bif195, and the TEER response was measured hourly for 16 h (Figure 6A). During the first 6 h after bacterial exposure, the TEER readings increased continuously, whereas after 6 h, the curve levelled out and stayed constant at approximately 140% of the baseline readings. TEER area under the curve (AUC) comparisons showed significantly higher AUC TEER for the Bif195 compared to the control medium (Figure 6B).

## Discussion

In this study, we aimed to investigate the effects of the probiotic strain *Bifidobacterium breve*, Bif195 on IBS-D by assessing symptoms associated with the disorder via validated scoring systems. The IBS–SSS score and the IBS–QoL score were used to evaluate the effects of Bif195, together with other independent symptomatic outcomes, which made it possible to address a broad range of frequent somatic and mental health-related issues. However, no effects of Bif195 on the primary or secondary outcomes were found. Among the three explorative outcomes, we observed one statistically significant effect of Bif195: a positive effect on fatigue scores.

It has previously been hypothesised that increased gut permeability may be one of the key reasons for fatigue development in patients with IBS (5, 24), possibly due to gut–brain axis involvement facilitated by neurohumoral communication between the brain and GI tract (25). For the disorders fibromyalgia and myalgic encephalomyelitis/chronic fatigue syndrome, growing evidence suggests an association between increased gut permeability and fatigue due to dysfunction of the intestinal mucosal barrier and the cascade of immune responses caused thereby (26). Based on the impact on fatigue in the current study, we conducted an experiment where the influence of Bif195 on epithelial integrity was explored in a well-established Caco-2 cell model (27, 28). Bif195 significantly increased TEER over time, which could suggest that supplementation with Bif195 positively affects the permeability of the intestine. The species *B. breve* is acknowledged to produce metabolites, such as acetate, and aromatic amino acids involved in the maturation and strengthening of the epithelial barrier (28, 29). Further, in previous studies, a highly conserved gene locus encoding a Type IVb tight adherence E (TadE) pili, acknowledged to accelerate epithelial cell proliferation, was identified in *B. breve* strains (30, 31), which could also play a key role in improving barrier integrity.

The effect of Bif195 on intestinal permeability has already been investigated in a healthy population via an exercise-induced permeability model without significant outcomes (32), most likely due to the rapid spontaneous recovery of the intestinal barrier in these healthy subjects following strenuous exercise. Nevertheless, investigation of barrier integrity in a population with IBS-D could potentially provide different results due to differences in the gut microbiota and underlying inflammatory conditions. For future perspectives, the association described in the literature between increased gut permeability and fatigue adds to the relevance of further investigations into the effect of Bif195 on gut permeability in a population with IBS-D.

We measured fatigue using the 11-point NRS and found a mean change in fatigue scores in the Bif195 group at −0.9 (range −4.7 to 2.9) from baseline to the end of intervention. Even though this was a statistically significant score reduction compared to the placebo group, we observed no overall improvement in quality of life among participants consuming Bif195 compared to placebo. This could be due to the low weight of fatigue-related questions within the IBS-QoL. Of a total of 34 questions, only 3 can be interpreted as fatigue-related: “Q18. I feel I get less done because of my bowel problems,” “Q22. I have to avoid strenuous activity because of my bowel problems,” and “Q25. I feel sluggish because of my bowel problems” (16). For two of these items, Q18 and Q22, it is debatable whether they are solely fatigue-related or if the level of agreement, stated by study participants, also depends on other factors such as abdominal pain, frequent toilet visits, or urgency. Fatigue was an exploratory endpoint and therefore not adjusted for multiple testing.

Another explorative outcome of this study was gut microbiome analyses. In the Bif195 group, richness and beta diversity of the gut microbiome, and the relative abundance of *B. breve* were significantly different after 8 weeks of treatment compared to baseline. However, the differential abundance analysis of the Bif195 group showed that the treatment did not result in engraftment of the probiotic strain in the gut microbiota of the host. Thus, after termination of the treatment, the relative abundance of *B. breve* returned to baseline levels, correlating with fatigue scores that did not differ significantly between baseline and the follow-up visit.

Ongoing discussions debate the dose and duration of a probiotic treatment to successfully affect the outcomes of clinical studies. A recent meta-analysis stated that with the existing data from clinical trials using different probiotic strains, experimental outcomes, doses, and duration of interventions, it is difficult to draw standardised conclusions (33). However, Yang et al. (33) distinguished between shorter treatment duration and long-term use of probiotics with a cut-off at 8 weeks, indicating that interventions of 8 weeks or longer are preferable for the probiotic strain to improve chances of colonisation of the gut and successfully improve IBS symptoms.

Previous trials with Bif195 in healthy populations, such as NSAID-models and an intestinal permeability model, have shown that Bif195 at various doses (15*10^9^–100*10^9^ CFU/day) was safe and well-tolerated (13, 32, 34). In general, the safety data based on reported adverse events from the present study in a population of patients with IBS-D did not differ from previous results establishing Bif195 as being safe.

One of the strengths of this study is the collection of faecal samples, which made it possible to confirm a high study product compliance in the Bif195 group due to the increased relative abundance of *B. breve* and the detection of Bif195 DNA by qPCR. All participants included in the PPS had a study product intake of >80, and 80.8% of the participants consumed more than 90% of the product. The high study compliance among the participants adds strength to the outcomes of the study.

Consumption of antibiotics and probiotics for 4 weeks prior to inclusion and during the study period was not allowed. Participants were also asked not to induce major lifestyle changes during the study. However, we did not ask participants to keep a diary of their food intake, nor did we have any policies regarding the intake of fermented food, dietary products, fibres, etc. This may be a limitation of the study.

Other limitations of this study include not correcting for multiple testing in the exploratory analyses and the use of the NRS fatigue scale, which is not validated for IBS populations.

In conclusion, supplementation for 8 weeks with Bif195 did not affect the primary endpoint IBS-SSS scores or any of the secondary outcomes compared to placebo in a population of men and women with IBS-D. However, the results showed that Bif195 lowered fatigue scores compared to placebo. A possible mechanism was explored in an *in vitro* experiment showing that Bif195 enhanced barrier integrity in Caco-2 monolayers.

Supplementation with Bif195 was reflected in the participants’ gut microbiome, resulting in changes in richness, beta diversity, and relative abundance of *B. breve* after 8 weeks. Yet the changes were transient, as none of the outcomes were significantly different from baseline at follow-up 8 weeks after the termination of treatment. The study showed that Bif195 was well-tolerated and safe to use in a group of IBS-D patients.

## Data Availability

The datasets presented in this study can be found in online repositories. The names of the repository/repositories and accession number(s) can be found at: https://www.ncbi.nlm.nih.gov/, PRJNA1269031.
