# Supplementary material for: Eight weeks of treatment with probiotic Bifidobacterium breve, Bif195 lowers fatigue scores in patients with diarrhoea-predominant irritable bowel syndrome: results from a randomised, clinical trial
Source: Front Nutr. 2026 Jan 21;12:1701341. doi: 10.3389/fnut.2025.1701341 (PMC12869991; doi:10.3389/fnut.2025.1701341)
Supplement: Supplementary file 1 [file Supplementary_file_1.docx]

**Supplementary Figures and Tables**

| Supplementary Table 1. Explorative outcomes: Changes in the Bif195 group and the placebo group after eight weeks of treatment | | | | |
| --- | --- | --- | --- | --- |
| Outcomes | Overall  (n=52) | Bif195  (n=25) | Placebo  (n=27) | **p-value* |
| Fatigue score  n  mean change (SD) | 52  -0.4 (1.76) | 25  -0.9 (1.74) | 27  0.1 (1.67) | **0.016** |
| Stool frequency  n  mean change (SD) | 52  -0.2 (1.32) | 25  -0.1 (1.60) | 27  -0.4 (1.02) | 0.409 |
| Urgency  n  mean change (SD) | 40  -0.1 (1.41) | 19  0.3 (1.67) | 21  -0.5 (1.05) | 0.178 |
| n = number of subjects within the population  SD = standard deviation  *ANCOVA analysis of the mean delta values (V3-V1), with baseline values as covariate | | | | |


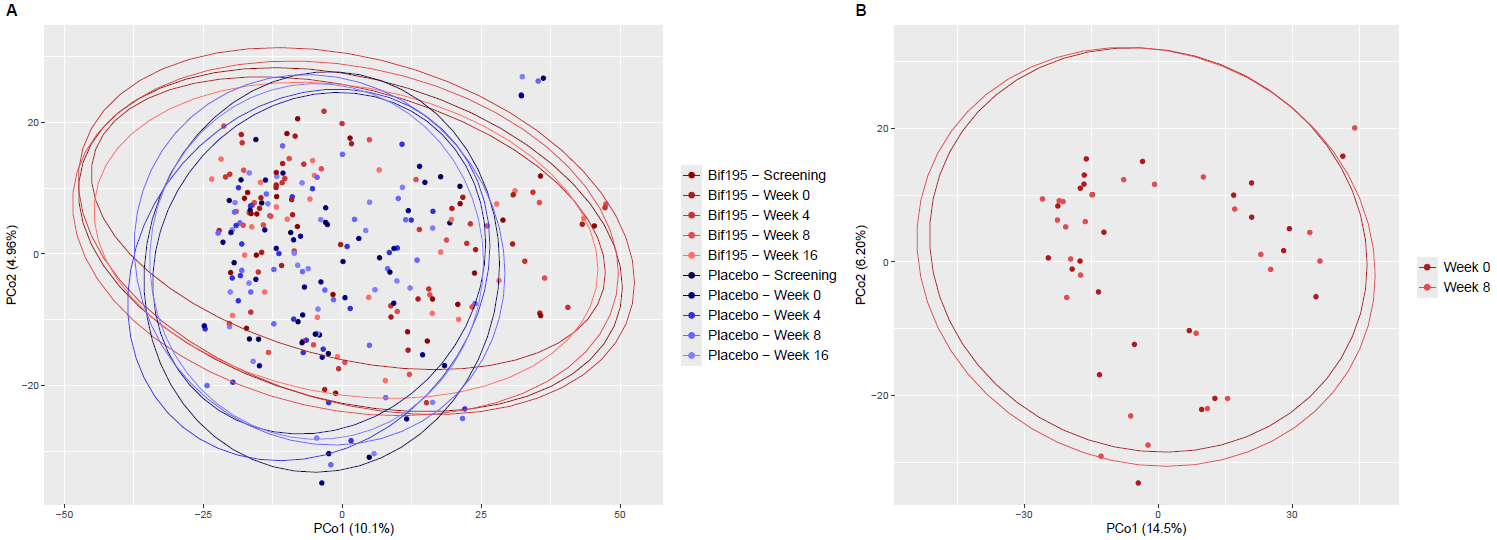


**Supplementary Figure 1.** Beta diversity divided into study visits in the Bif195 group and placebo group (A) and the beta diversity in the Bif195 group with comparison of data from baseline at week 0 and after eight weeks of treatment (B). Beta diversity was significantly different between baseline and after eight weeks of treatment in the Bif195 group, *p*=0.015.
